# Serological differentiation between COVID-19 and SARS infections

**DOI:** 10.1080/22221751.2020.1780951

**Published:** 2020-07-07

**Authors:** Wan Ni Chia, Chee Wah Tan, Randy Foo, Adrian Eng Zheng Kang, Yilong Peng, Velraj Sivalingam, Charles Tiu, Xin Mei Ong, Feng Zhu, Barnaby E. Young, Mark I.-C. Chen, Yee-Joo Tan, David C. Lye, Danielle E. Anderson, Lin-Fa Wang

**Affiliations:** a Programme in Emerging Infectious Diseases, Duke-NUS Medical School, Singapore, Singapore; b National Centre for Infectious Diseases, Singapore, Singapore; c Tan Tock Seng Hospital, Singapore, Singapore; d Lee Kong Chian School of Medicine, Nanyang Technological University, Singapore, Singapore; e Saw Swee Hock School of Public Health, National University of Singapore, Singapore, Singapore; f Department of Microbiology and Immunology, Yong Loo Lin School of Medicine, National University Health System (NUHS), National University of Singapore, Singapore, Singapore; g Institute of Molecular and Cell Biology (IMCB), A*STAR (Agency for Science, Technology and Research), Singapore, Singapore; h Yong Loo Lin School of Medicine, National University of Singapore, Singapore, Singapore

**Keywords:** SARS, COVID-19, SARS-CoV-2, antibody, serology

## Abstract

In response to the coronavirus disease 2019 (COVID-19) outbreak, caused by SARS-CoV-2, multiple diagnostic tests are required for acute disease diagnosis, contact tracing, monitoring asymptomatic infection rates and assessing herd immunity. While PCR remains the frontline test of choice in the acute diagnostic setting, serological tests are urgently needed. Unlike PCR tests which are highly specific, cross-reactivity is a major challenge for COVID-19 antibody tests considering there are six other coronaviruses known to infect humans. SARS-CoV is genetically related to SARS-CoV-2 sharing approximately 80% sequence identity and both belong to the species *SARS related coronavirus* in the genus *Betacoronavirus* of family *Coronaviridae*. We developed and compared the performance of four different serological tests to comprehensively assess the cross-reactivity between COVID-19 and SARS patient sera. There is significant cross-reactivity when N protein of either virus is used. The S1 or RBD regions from the spike (S) protein offers better specificity. Amongst the different platforms, capture ELISA performed best. We found that SARS survivors all have significant levels of antibodies remaining in their blood 17 years after infection. Anti-N antibodies waned more than anti-RBD antibodies, and the latter is known to play a more important role in providing protective immunity.

## Introduction

In December 2019, a novel coronavirus first emerged in Hubei province in China and the virus was identified as 2019-nCoV, later designated SARS-CoV-2 [1,2]. The associated disease is referred to as COVID-19. On 30 January 2020, the World Health Organization declared COVID-19 a Public Health Emergency of International Concern and declared a pandemic on 11 March 2020. As of 1 June 2020, the COVID-19 outbreak has led to 6,057,853 confirmed cases and 371,166 deaths globally [3]. The virus demonstrated efficient human-to-human transmission and the epicentre has shifted from mainland China to Europe and USA, and then to South America [3].

Laboratory diagnostic tests play a pivotal role for any infectious disease outbreak response, which is also true for COVID-19. Within days of the SARS-CoV-2 genome release into the public domain, multiple PCR tests were rapidly developed and implemented at the frontline for diagnosis of acute pneumonia patients in China and globally [4]. When the outbreak progressed further, it was evident that PCR tests alone could not meet the other needs of the COVID-19 response, such as retrospective contact tracing, investigation of asymptomatic infection rate and assessment of herd immunity [5].

There is an urgent need for serology or antibody tests. Research laboratories and pharmaceutical companies are racing to produce tests that can detect COVID-19 infection with sufficient specificity and sensitivity [5]. In addition to SARS-CoV-2, there are six other coronaviruses (OC43, 229E, SARS-CoV, NL63, HKU1 and MERS-CoV) known to infect humans [6–10]. This presents a major challenge when aiming for test specificity. Although the possibility of antibody cross-reactivity among all these human coronaviruses (hCoVs) exists, SARS-CoV presents the highest chance of cross-reactivity with SARS-CoV-2 because of the close phylogenetic relationship and the high genome and protein sequences identity [11]. Determining the level of cross-reactivity between COVID-19 and SARS antibodies is especially important for countries like China and Singapore, which were affected by both outbreaks [12,13]. The current study focused on the development of serologic tests which can reliably differentiate COVID-19 infection from SARS infection.

The major viral antigens used most frequently in antibody tests for coronavirus infections are two of the four major structural proteins, the nucleocapsid protein (N) and the spike protein (S) [10]. The CoV S protein is a large envelope protein, and is 1,273 aa long for SARS-CoV-2 [14]. The S protein is cleaved by host protease into two subunits, the N-terminal S1 subunit (aa 1-685) responsible for receptor binding and the C-terminal S2 subunit, responsible for membrane fusion. The receptor-binding domain (RBD) is located at the C-terminal region (aa 319-591) of the S1 subunit, and recombinant RBD alone has been shown to be sufficient to bind the cell entry receptor, angiotensin-converting enzyme 2 (ACE2) [15,16].

In this study we examined the performance of N, S1 and RBD proteins from SARS-CoV-2 and SARS-CoV in four different test platforms. Our results show that the RBD protein provides the best specificity, whereas the N protein of either virus is not suitable to detect virus-specific antibodies due to very high level of cross-reactivity. We demonstrated that capture ELISA can further enhance test specificity and the assay format can be easily adapted to studying isotype- (IgG, IgM, etc.) and subtype-specific antibody responses for more basic research needs. Unexpectedly, we discovered that the N-specific antibodies waned faster than the RBD-specific antibodies in convalescent sera obtained from SARS survivors, seventeen years after infection. The significance and the mechanism behind this observation warrants further investigation in the future.

## Materials and methods

### Cells and virus

Human embryonic kidney (HEK293T) cells (ATCC# CRL-3216) were maintained in Dulbecco’s modified Eagle Medium (DMEM) supplemented with 10% foetal bovine serum. The first SARS-CoV-2 isolate from Singapore, BetaCoV/Singapore/2/2020 (Accession ID EPI_ISL_406973), was used for virus neutralization test using Vero-E6 cells as described previously [13].

### Panels of human sera used in this study

COVID-19 patient sera used in this study was from the Singapore PROTECT cohort as described [13]. Serum samples were collected 14–32 days post symptom onset. Sera from recovered SARS patients from 2003 were as previously described [17]. For SARS recall sampling in 2020, we contacted and then obtained blood from consenting individuals previously admitted for SARS (ethics reference NHG DSRB E 2020/00091). None of these individuals had confirmed or suspected exposure to SARS-CoV-2, according to the national contact tracing data available. Negative control sera were obtained from residual serum samples from previous unrelated studies prior to December 2019.

### Luciferase immunoprecipitation system (LIPS)

Published methods [18,19] were followed in this study. The N genes of SARS-CoV and SARS-CoV-2 were cloned in-frame with the Renilla luciferase gene (Rluc) and flag tag in the pREN2 vector [20]. The plasmids were then transfected into COS-1 cells using FuGENE^®^ 6 (Promega) and cells were collected 48 h post-transfection. The cells were lysed and luciferase assay (Promega) was performed following manufacturer’s recommendations. To determine serum reactivity to SARS-CoV or SARS-2-CoV N proteins, 1 µl of serum was incubated with cell lysate corresponding to 5 million light units (LU) of Rluc-N fusion protein and 6 µl of 30% Protein A/G agarose slurry (Invitrogen) for 2 h at 4°C. After extensive washing to remove unbound luciferase-tagged antigens, the captured luciferase was determined using luciferase substrate (Promega).

### Luminex assay

All recombinant proteins used in this study were His-tagged for normalization. SARS-CoV-2 and SARS-CoV N proteins were purified from pcDNA3.1-SARS-CoV-2 N or pDual-GC-SARS-CoV N transfected HEK293 T cells using Ni Sepharose affinity chromatography (GE Healthcare). SARS-CoV-2 S1 and SARS-CoV S1 were obtained from Sino Biological. SARS-CoV-2 RBD and SARS-CoV RBD were custom produced by GenScript. The amino acid sequence of these recombinant proteins are given in Supplementary Table 2. 25 μg of each antigen was coupled onto MagPlex-c microsphere (Luminex) using xMAP antibody coupling kit (Luminex) according to the manufacturer’s instructions. To assess the antibody reactivity, 1250 beads/antigen were incubated with various serum samples at 1:100 dilution in PBS containing 1% BSA for an hour at 37°C with agitation. The bound antibodies were detected with goat anti-human IgG, PE (eBioscience) at 1:1000 dilution. The level of antibody binding was determined using the Luminex MAGPIX system. The readings were normalised by dividing with the readings obtained from the anti-His tag antibody (Thermo Scientific) and presented as net mean fluorescence intensity (MFI).

### Indirect ELISA

96-well Maxisorp plates (Nunc) were coated with 2 µg/ml of SARS-CoV-2 RBD protein (Genscript) in bicarbonate buffer overnight at 4°C. Wells were blocked using BD OptEIA assay diluent (BD) for 1 h at 37°C. Heat-inactivated sera were depleted for serum IgG using Gullsorb™ Human IgG Inactivation Reagent (Meridian Bioscience) as per manufacturer’s recommendations. Depleted and undepleted sera were then further diluted to a final concentration of 1:200, added into ELISA microwells and incubated for 1 h at 37°C. Following extensive washing, anti-human IgM-HRP (Life Technologies) or anti-human IgG-HRP (Santa Cruz) diluted 1:10,000 was added and incubated for 30 min at 37°C. The chromogenic reaction was quantified following the addition of TMB substrate (Invitrogen) and stop solution (KPL SeraCare). The absorbance of the samples was measured at 450 nm and the background at 570 nm. The results are presented as the OD difference of 450 and 570 nm.

### Capture ELISA

96-well Maxisorp plates (Nunc) were coated with 10 µg/ml of anti-human IgM (SeraCare) or anti-human IgG (Jackson labs) in bicarbonate buffer overnight at 4°C. Wells were blocked using BD OptEIA assay diluent (BD) for 1 h at 37°C and heat-inactivated sera diluted 1:50 were next added and incubated for 1 h at 37°C. Following extensive washing, SARS-CoV-2-HRP (GenScript) diluted 4 µg/ml was added and incubated for 30 min at 37°C. Chromogenic reaction was quantified following the addition of TMB substrate (Invitrogen) and stop solution (KPL SeraCare). The absorbance of the samples was measured at 450 nm and the background at 570 nm. Negative controls consisting of 37 naïve human sera were added in the assay. The results are presented as fold change over average reading of negative controls.

### Virus neutralization test (VNT)

For VNT, 50 μl of two-fold serial diluted sera were pre-incubated with 50 μl of 1000 TCID_50_/ml of SARS-CoV-2 in 5% FBS-DMEM for 90 min at 37°C. The virus-serum mixtures were then added onto monolayers of Vero-E6 cells for 60 min at 37°C. At 1 h post infection, the inoculum was removed and infected cells were washed once with DMEM. Cells were then replenished with 5% FBS-DMEM and the neutralization titers were determined at 4 days post infection.

### Statistical analysis

Statistical analysis was perform using GraphPad Prism software. Statistical analysis for LIPS, Luminex and ELISA results were performed using Kruskal–Wallis test to compare multiple groups followed by Dunn’s multiple comparisons test. Data were considered significant if * *P*<0.05, **P<0.01, ***P<0.001, ****P<0.0001.

### Phylogenetic analysis

Nucleocapsid and spike protein sequences of all seven human coronaviruses were retrieved from NCBI (Accession numbers see Supplementary Table 1). The protein alignments were performed by Clustal Omega (v.1.2.2) on Geneious Prime (v.2020.1.1). Maximum-Likelihood phylogenetic trees were created by using PhyML with Smart Model Selection and 1000 bootstraps [21].

## Results

### N protein-based luciferase immunoprecipitation system (LIPS) assay displayed high cross reactivity

**Figure 1.**
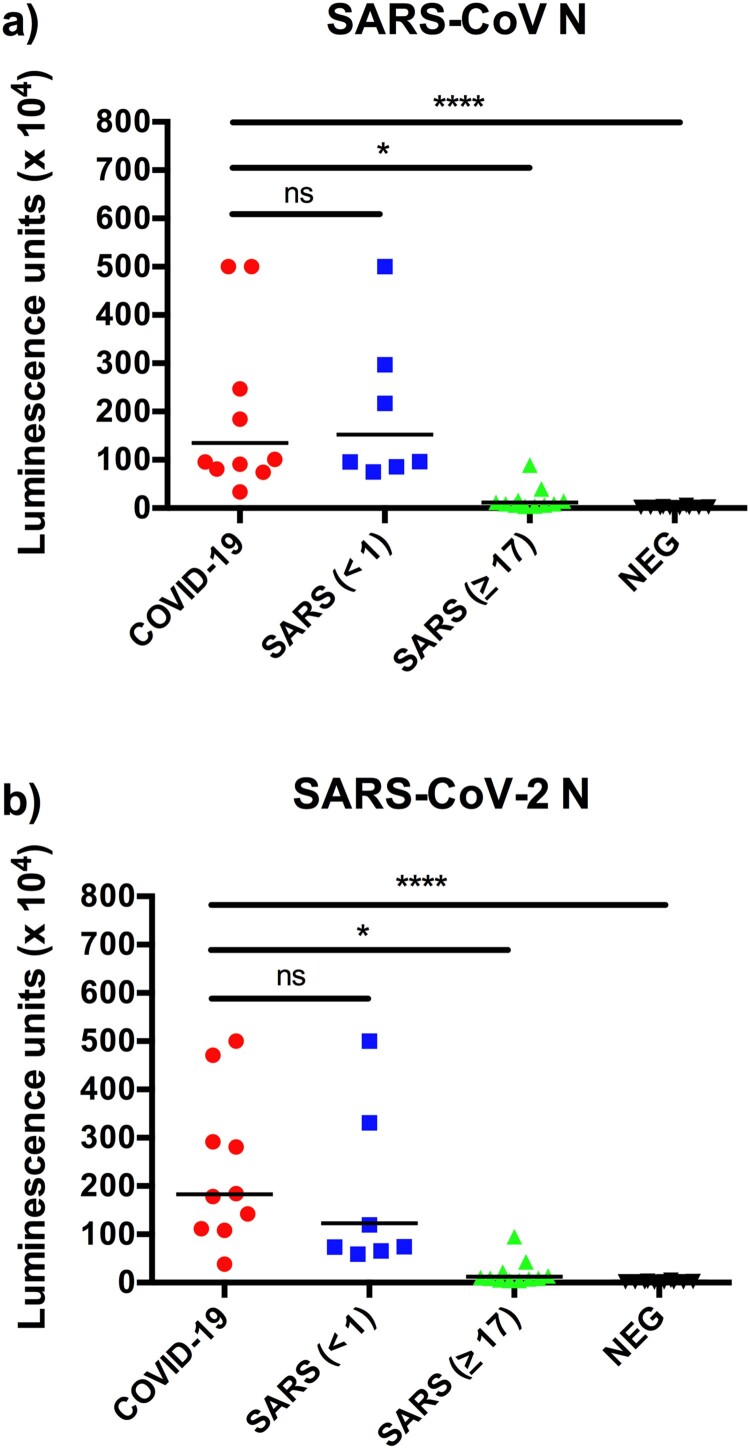
Rapid detection of N-specific antibodies using LIPS. Data presented are luminescence units against N proteins of SARS-CoV (a) and SARS-CoV-2 (b). The SARS sera were divided into those collected in 2003 (<1 year) or 2020 (≥17 years).

**Figure 2.**
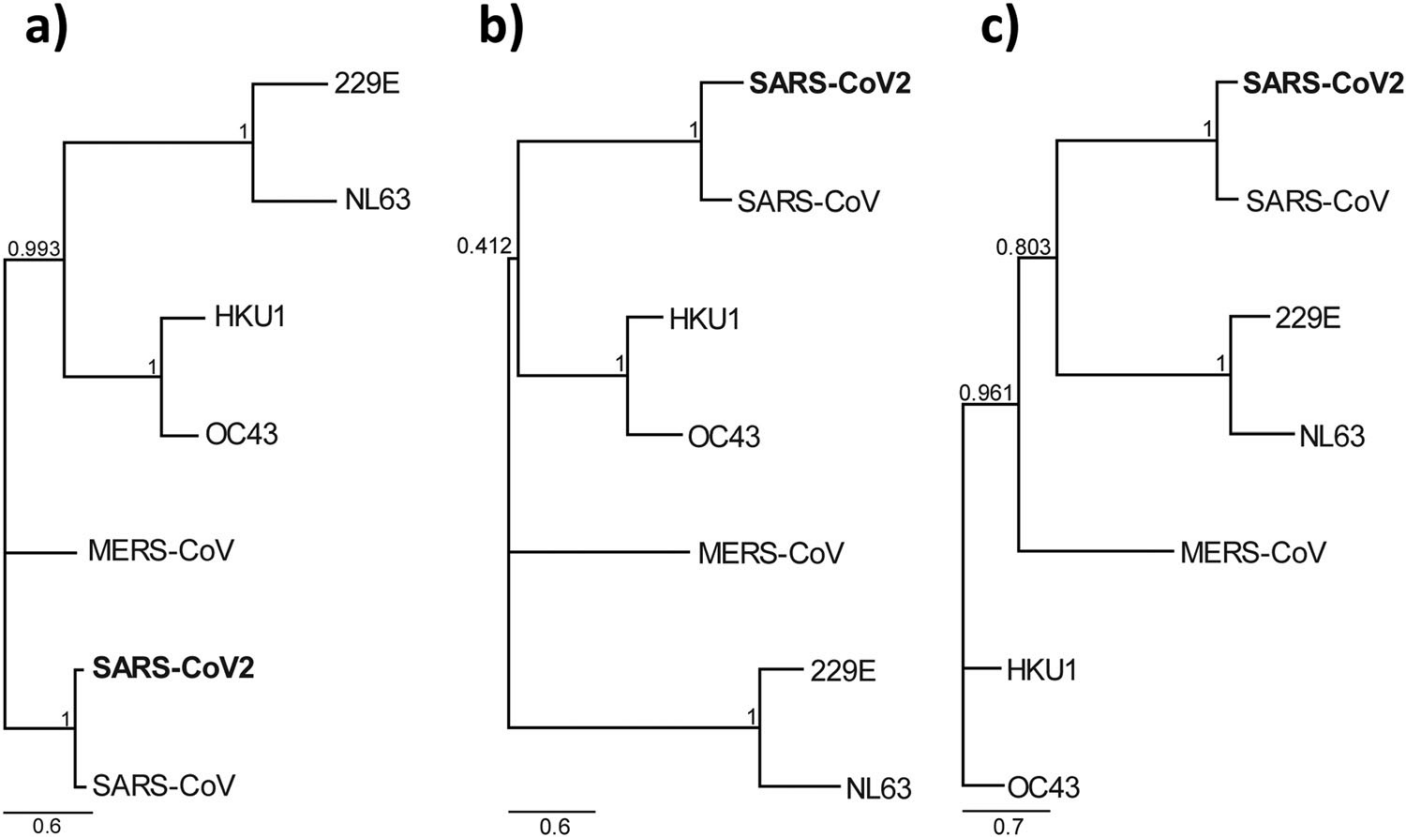
Phylogeny based on the three different proteins of seven known human coronaviruses. Maximum likelihood phylogenetic tree based on amino acid sequences of (a) Nucleocapsid protein (b) S1 protein and (c) RBD of human coronaviruses with 1000 bootstrap replicates. Support values are indicated at nodes. The scale bars represent substitutions per amino acid position.

The luciferase immunoprecipitation (LIPS) assay is most suited for very rapid development of serologic test from genome sequence information alone [19,20]. In the past, we applied this platform to the successful investigation of potential zoonotic transmission of a HKU2-related bat coronavirus during a major disease outbreak in pigs in 2017 [19]. Within a week of receiving the SARS-CoV-2 N gene, we developed a LIPS assay to detect antibodies to the N proteins of SARS-CoV and SARS-CoV-2. The testing panel was composed of the first 10 available sera from COVID-19 confirmed patients, 18 SARS sera and 10 negative sera. While the detection sensitivity was good, the specificity was poor as there was high level of two-way cross-reaction between sera from COVID-19 and SARS patients (Figure 1). The high cross-reactivity between the two N proteins is not unexpected as both are highly related as indicated by phylogeny (Figure 2) and protein sequence identity analysis (Table 1). No significant N-specific antibodies were detected in SARS sera collected 17 years after infection. The LIPS-based assay was used as a rapid response tool, but with the high cross-reactivity observed between COVID-19 and SARS sera, we decided to not pursue LIPS in further analysis.

**Table 1.** Percentage sequence identity to SARS-CoV-2 proteins.

Viruses	% identity		
	*N*	S1	RBD
SARS-CoV	89.29	62.87	74.61
MERS-CoV	45.11	16.87	13.95
OC43	29.66	16.62	16.33
229E	21.69	9.67	10.49
NL63	23.19	9.26	8.61
HKU1	27.96	16.69	14.44

### Multiplex comparative analysis using Luminex with six recombinant proteins

**Figure 3.**
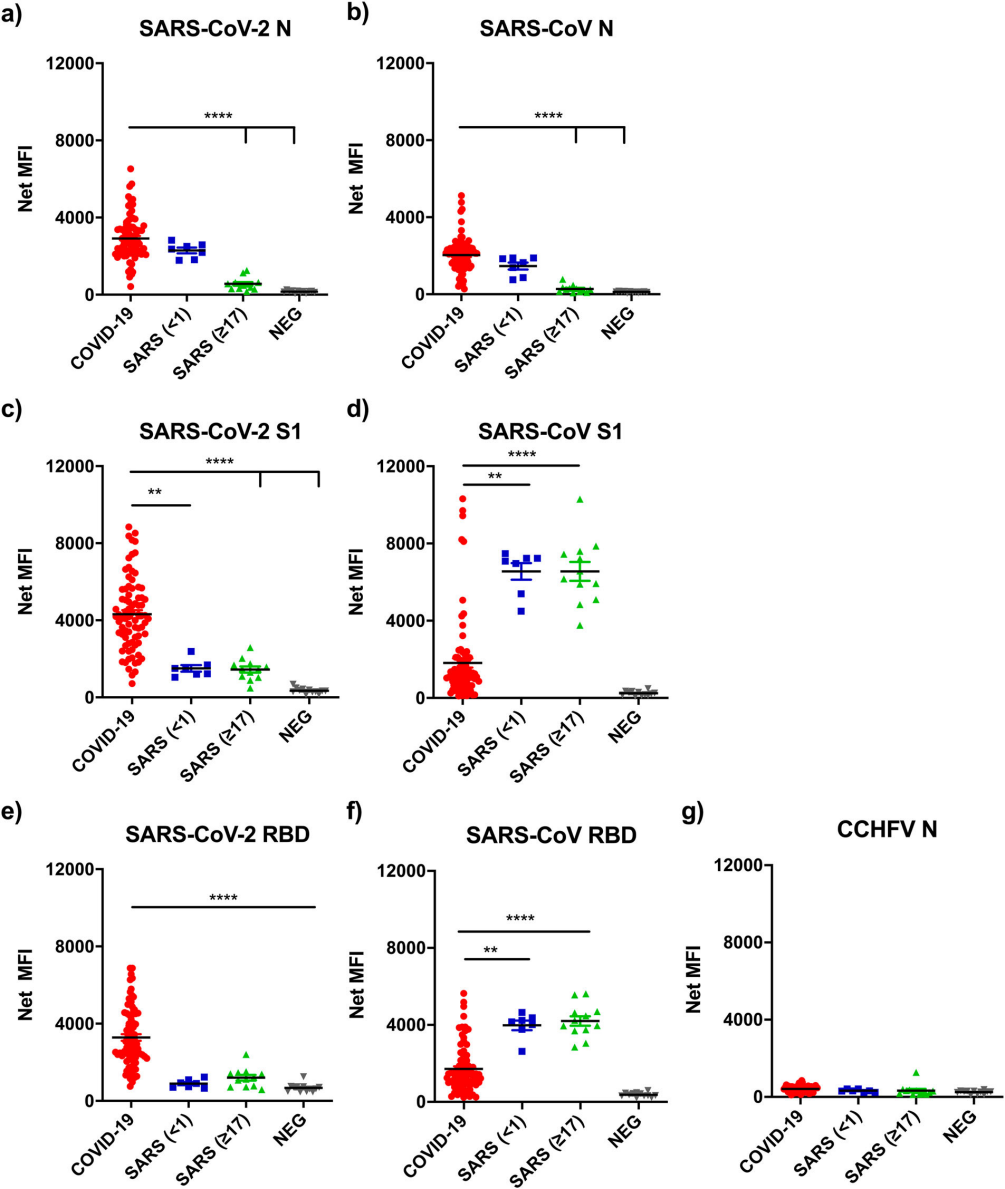
Analysis of antibody binding from COVID-19 and SARS sera against seven recombinant proteins. Show on the *Y*-axis are net mean florescence (MFI) units normalised with those obtained from anti-His antibody readings for each protein (see Methods for detail). The serum panels includes 37 healthy controls, 74 sera from PCR positive COVID-19 patients, and 18 recovered SARS patients. The SARS sera were divided into those sampled in 2003 (<1 year, *n*=7) or 2020 (≥17 years *n*=11).

While the two N proteins are close to 90% identical between SARS-CoV-2 and SARS-CoV, the S1 and RBD protein sequences only share 62%–74% identity, indicating the S protein is more virus-specific (Table 1). We employed the multiplex Luminex platform [22] to better assess cross-reactivity between different proteins in a single assay, avoiding inter-assay variation. Seven antigens were included; N, S1, and RBD of both SARS-CoV and SARS-CoV-2, and N of Crimean-Congo haemorrhagic fever virus (CCHFV) [23] as a negative control. All recombinant antigens were His-tagged. Antigens were conjugated onto the MagPlex-c microsphere at fixed concentration of 50 µg/ml and further quantified by anti-His mAb binding analysis. With more COVID-19 sera available by the time the Luminex assay was developed, we expanded the serum panels to include 30 negative, 74 COVID-19 and 18 SARS sera, which were divided into two groups, serum sampled in 2003 within 1 year of the infection (*n*=7) and in 2020, 17 years post infection (*n*=11). These same serum panels were used for all subsequent analyses in this study. The assay is highly specific. Firstly, there was no significant binding on the negative control antigen CCHFV N (Figure 3(g)); and secondly, none of the negative control sera had significant reactivity against any of the six recombinant antigens included in this study (Figure 3(a–f)).

For N protein, similar results to those from LIPS were obtained. There was significant two-way cross-reactivity between COVID-19 and SARS 2003 sera (Figure 3(a,b)). In contrast, binding to the S1 or RBD proteins was more virus-specific, although there was still some level of cross-reactivity, it was more prominent for COVID-19 sera to react with SARS-CoV antigens rather than vice versa (Figure 3(c–f)). It was further confirmed that SARS 2020 sera had very low or no N-specific antibodies (Figure 3(a,b). In contrast, SARS 2020 sera had similar levels of S1- or RBD-specific reactivity to those from 2003 (Figure 3(c–f)). Although both S1 and RBD showed higher specificity than the N protein, RBD was chosen for subsequent studies as RBD is expected to better correlate with virus-neutralizing antibodies. Several published studies confirm that RBD is the most specific antigen for detection of COVID-19 antibodies [24,25].

### Indirect ELISA based on the SARS-CoV-2 RBD protein

**Figure 4.**
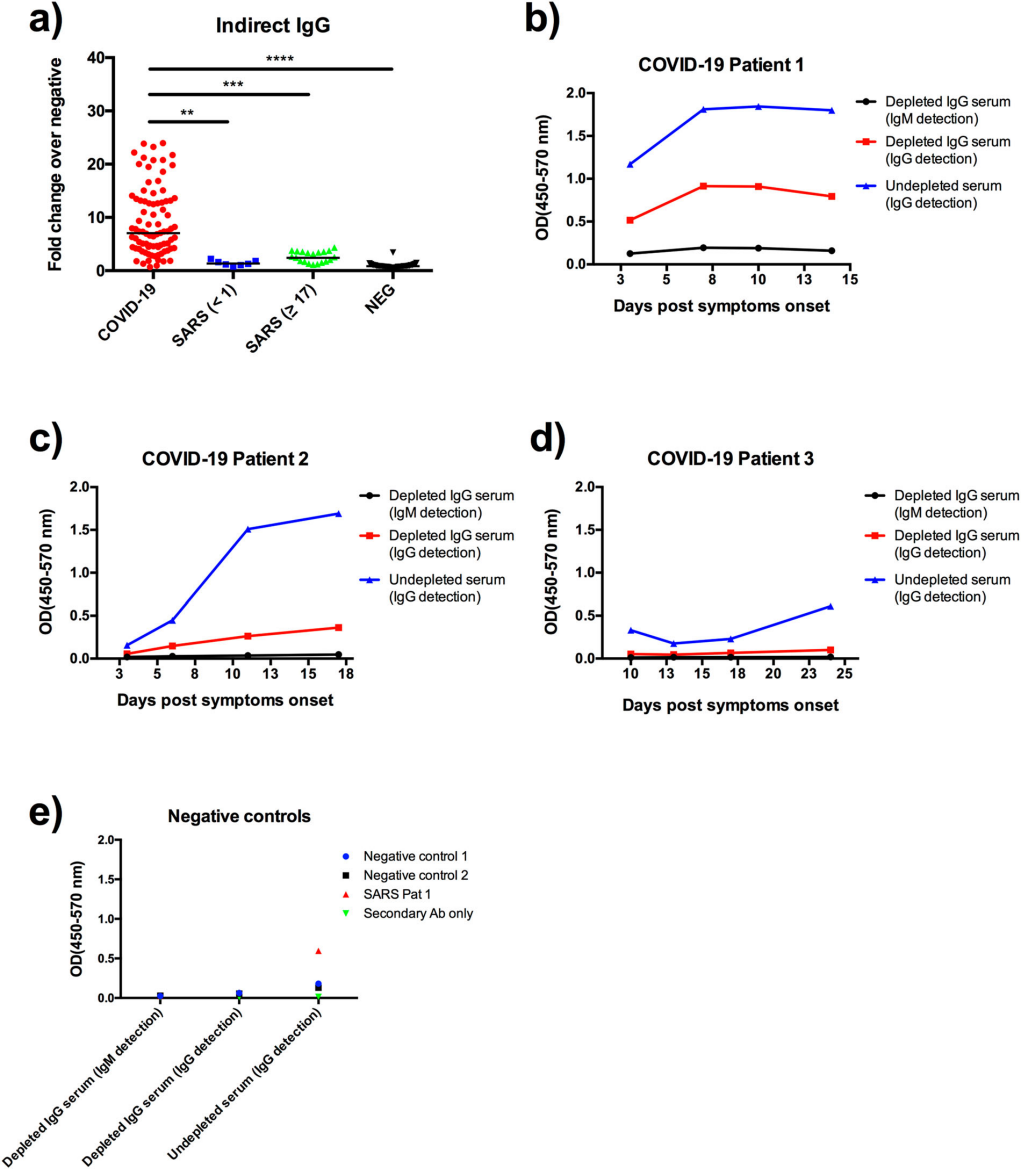
Detection of anti-RBD IgG and IgM antibodies by indirect ELISA. (a) IgG data obtained from the same serum panels as those in Figure 3. IgM testing with or without IgG depletion from three representative COVID-19 patient sera known to have high (b), medium (c) and low (d) IgG antibody levels. Also included are two healthy controls and one SARS patient serum (e). Data are presented as fold of change (Fc) over the average reading of negative controls.

Although multiplex Luminex provided a good holistic overview of the two-way cross-reactivity with the six recombinant proteins from the two viruses, it is not realistic to implement this assay as a frontline screening test. We next assessed an indirect ELISA assay format using the SARS-CoV-2 RBD with the rationale provided above. Both IgG and IgM binding were examined in this study. The IgG data (Figure 4(a)) demonstrated similar performance as the Luminex data based on SARS-CoV-2 RBD (Figure 3(e)). However, initial IgM testing revealed a high background which make data interpretation almost impossible (data not shown). An IgG depletion step was included to reduce background with three representative patient sera with weak, moderate and strong IgG responses against SARS-CoV-2. The data demonstrate that while the IgG binding is specific and sensitive, there is poor detection of IgM with or without IgG depletion treatment.

### Capture ELISA using a horseradish-conjugated RBD protein (HRP-RBD)

**Figure 5.**
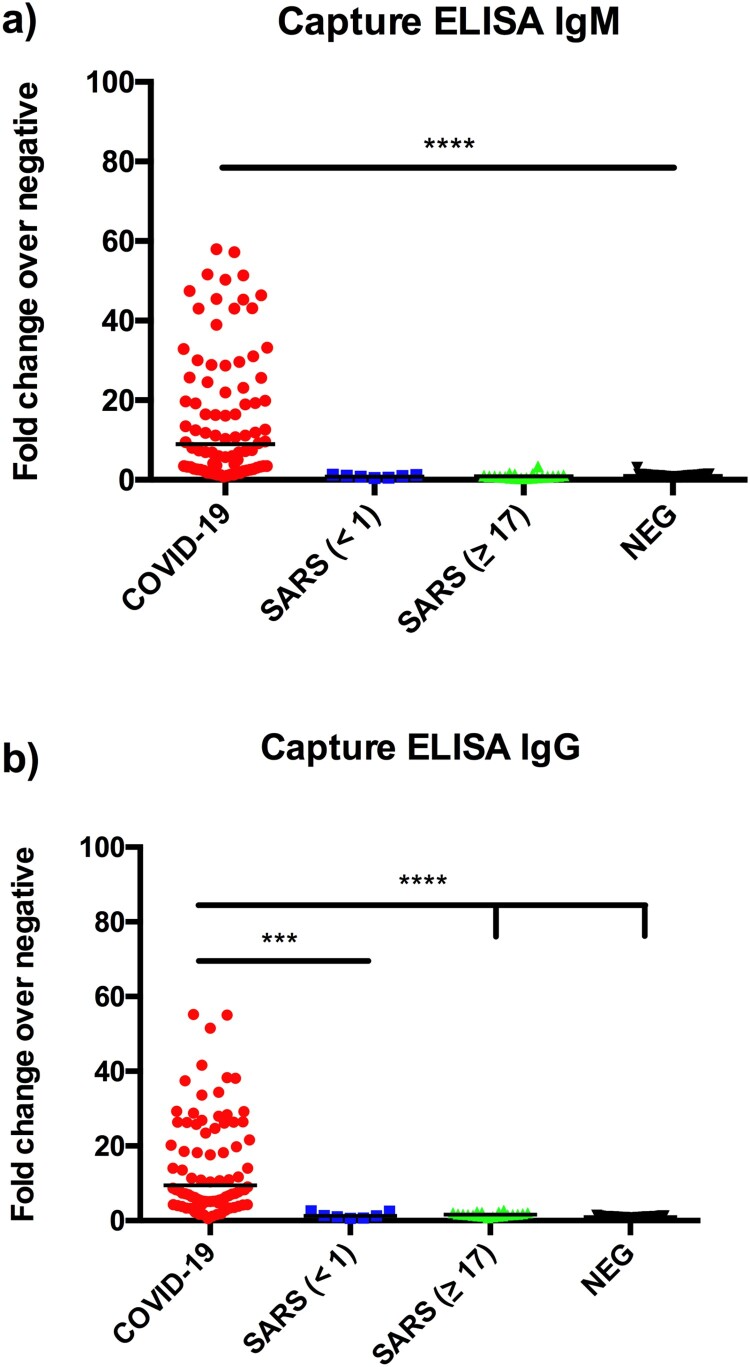
Detection of anti-RBD IgG (a) and IgM (b) antibodies by capture ELISA. Data are presented as fold of change (Fc) over the average reading of negative controls. The same serum panels as in Figure 3 were used in this study.

To further improve assay specificity, we established a capture ELISA using a horseradish peroxidase-conjugated RBD protein (HRP-RBD) from SARS-CoV-2. The ELISA plate was precoated with human isotype-specific anti-hIgG or anti-hIgM antibodies. After adding test human sera and washing, HRP-RBD was added, followed by washing and colour development. The results illustrated a significant improvement for IgM detection over the indirect ELISA (Figure 5), achieving a specificity of 100% and sensitivity of 96% for all PCR-positive patients.

Prior to the development of the capture ELISA, the indirect IgG ELISA was used to screen healthy controls and samples from contact tracing and other investigations. In addition to clear negative or positive samples, we also found some mid-range positive samples. Five of these samples were tested by virus neutralization test (VNT) and found to be negative. When these samples were re-tested in the capture ELISA, they were also negative (Table 2), confirming superior specificity over the indirect ELISA.

**Table 2.** Comparison of IgG detection by indirect and capture ELISA* and validation with VNT.

Samples	Indirect IgG (Fc)	Capture IgG (Fc)	VNT
Positive 1	16.3	5.2	Positive
Positive 2	26.2	6.7	Positive
Positive 3	21.2	7.0	Positive
Positive 4	25.5	12.1	Positive
Suspected positive 1	15.2	0.8	Negative
Suspected positive 2	7.7	1.7	Negative
Suspected positive 3	7.5	1.5	Negative
Suspected positive 4	3.4	1.2	Negative
Suspected positive 5	10.0	0.9	Negative

*For ELISA readings, positive samples are defined as those have fold of change (Fc) equal to or greater than 3.

## Discussion

When combating infectious disease outbreaks such as COVID-19, rapid development of diagnostic assays is a pivotal part of the response. Molecular tests, mainly PCR, are much faster to develop, but they are unable to meet all the needs of an outbreak response. Serology remains an integral part of the overall response and can complement PCR-based diagnosis by confirming antibody response during the early stage of the infection, which is especially true for IgM antibodies [26].

More importantly, serology plays a key role in contact tracing/epidemiology and assessment of sero-prevalence and longevity of protective immunity. This is especially important in the context of herd immunity for COVID-19 as, unlike the SARS outbreak, the outbreak is not expected to be eradicated in the foreseeable future [5]. Serology has also played an important role in the identification of natural reservoir host [27,28] or intermediate host [29–32] for emerging zoonotic viruses.

An ideal antibody test should have many or all of the following features: rapid (both for development and application), specific, sensitive, easy and safe to operate, inexpensive and portable. Unfortunately, there is no such “perfect” test. The pragmatic goal is therefore to develop a test that is “fit-for-purpose”. In this study, we have demonstrated that if speed of development is the goal, LIPS is the most suitable as a serologic test can be developed within days of the availability of a genome sequence. If multiplexity is the most desired feature, Luminex is ideal and highly reliable. On the other hand, indirect and capture ELISAs are inexpensive and can be established in most hospital diagnostic laboratories. In terms of detection limit, the indirect IgG ELISA has a better sensitivity at 0.457 than either capture IgG ELISA (2.384) or capture IgM ELISA (2.187). For Luminex, the detection limits vary a lot, depending on the viral antigen used, from 68 (SARS NP) to 1162 (SARS RBD), respectively. In future, it will be good to have a monoclonal antibody-based standard to more accurately determine detection limit for various COVID-19 serological assays.

Capture ELISA based on SARS-CoV-2 RBD is a user-friendly test, which is highly specific and sensitive. Since its establishment, we have tested a few hundred samples without detecting any false positive with a 100% specificity. The sensitivity presented in the manuscript was calculated by dividing the total number of positive samples by the total number of convalescent sera (sampled on 14–32 days post symptom onset) from PCR positive COVID-19 patients. However, as we know that not all COVID-19 patients seroconvert [33], the true sensitivity of our capture ELISA could be higher than 96%.

While we have focused our current study on the specific detection of IgG and IgM antibodies for SARS-CoV-2, the same test format can be easily adapted to other isotypes (IgA, IgE, IgD) or subtypes (IgG1-4) by changing the capture antibody used in the coating step. The HRP-RBD antigen is universal and so is the overall operating protocol. Similarly, the same capture ELISA can also be adapted to testing for antibodies to other antigen by replacing the HRP-RBD with another HRP-protein X, where X can be any other protein or domains of SARS-CoV-2.

Although we have not addressed the potential cross-reactivity of SARS-CoV-2 RBD with sera from humans infected with the other five known hCoVs (OC43, 229E, NL63, HKU1 and MERS-CoV), we are quite confident that this will not be an issue considering that even SARS patients with high levels of anti-SARS-CoV RBD antibodies did not pose any major cross-reactivity issues in our assays. This has been confirmed by published serological studies using SARS-CoV-2 RBD antigens [24,25].

The observation of S1- or RBD-specific antibodies lasting longer than N-specific antibodies in the sera of SARS survivors is of high interest. The biological significance and mechanistic characterization of this phenomenon are beyond the scope of this study but warrant further investigation, especially in the context of better understanding immunity longevity for recovered COVID-19 patients. It is also important to note that the current study only focused on antibody responses to N and RBD. It will be interesting to expand this study by comparing antibody responses in the 2003 and 2020 serum panels against other proteins of SARS-CoV, including the S2 subunit as it can also induce virus-specific immune responses, including neutralizing antibodies [33].

In conclusion, we have addressed the need for the use of different platforms and different viral antigens in an outbreak situation. We believe that the SARS-CoV-2 HRP-RBD based capture ELISA will be an effective tool for many applications requiring reliable, simple and specific antibody test for the continuing investigation of COVID-19 outbreak. The same capture format can be easily adapted to detect other isotype- or subtype-specific COVID-19 antibodies by simply changing the capture antibodies which are widely available commercially. Similarly, by changing the HRP-antigen, the same assay can be adapted to detect protein-specific antibodies to other proteins or ORFs of SARS-CoV-2.
